# Ginger intake suppresses neutrophil extracellular trap formation in autoimmune mice and healthy humans

**DOI:** 10.1172/jci.insight.172011

**Published:** 2023-09-22

**Authors:** Ramadan A. Ali, Valerie C. Minarchick, Miela Zahavi, Christine E. Rysenga, Kristin A. Sturm, Claire K. Hoy, Cyrus Sarosh, Jason S. Knight, M. Kristen Demoruelle

**Affiliations:** 1 Division of Rheumatology, Department of Internal Medicine, University of Michigan, Ann Arbor, Michigan, USA.; 2 Division of Rheumatology, University of Colorado School of Medicine, Aurora, Colorado, USA.

**Keywords:** Autoimmunity, Immunology, Autoimmune diseases, Neutrophils, Phosphodiesterases

## Abstract

We previously reported that treatment of mice with 6-gingerol, the most abundant phytochemical in ginger root, leads to phosphodiesterase inhibition that counteracts neutrophil hyperactivity in models of antiphospholipid syndrome (APS) and lupus. Here, we explored the extent to which oral intake of a whole-ginger extract would similarly impact neutrophils in both autoimmune mice and healthy humans. In vitro, a solubilized ginger extract was able to attenuate neutrophil extracellular trap formation (NETosis) by human neutrophils through a mechanism that was dependent upon the cyclic AMP–dependent kinase, protein kinase A. When mice with features of either APS or lupus were administered a ginger extract orally, they demonstrated reduced circulating NETs, as well as the tempering of other disease outcomes, such as large-vein thrombosis (APS) and autoantibody production (lupus). In a pilot clinical trial, which was validated in a second cohort, daily intake of a ginger supplement for 7 days by healthy volunteers boosted neutrophil cAMP, inhibited NETosis in response to disease-relevant stimuli, and reduced circulating plasma NET levels. In summary, this work demonstrates that ginger intake restrains neutrophil hyperactivity in autoimmune mouse models and that ginger consumption by healthy individuals makes their neutrophils more resistant to NETosis.

## Introduction

Chronic, incurable autoimmune diseases such as antiphospholipid syndrome (APS) and lupus are associated with significant morbidity, mortality, and health care costs (1–5). Lupus is the prototypical systemic autoimmune disease characterized by autoantibodies against nuclear components, which result in circulating immune complexes that deposit in and damage organs (6). APS, sometimes presenting in patients with lupus and sometimes as a standalone autoimmune disease, is associated with aberrant innate immune and vascular cell activation resulting in a markedly increased risk of thrombosis in vascular beds of all sizes (7). While these diseases demonstrate unique clinical phenotypes, there is convincing evidence that both are pathologically driven by a shared mechanism: dysfunctional and exaggerated neutrophil extracellular trap formation (termed NETosis). Through NETosis, neutrophils expel their nuclear chromatin in pro-inflammatory web-like structures that are decorated with potentially toxic granule-derived proteins (8, 9). Excessive NETosis propels inflammatory and thrombotic cascades, contributing to end organ damage over time and to the pathophysiology of many autoimmune diseases, including APS and lupus (10, 11).

Recent studies by our group and others have demonstrated that excessive NETosis has the potential to promote breaks in adaptive immune tolerance that result in durable autoantibody formation (12–14). At the same time, disease-associated autoantibodies drive further NETosis (15–18), thereby setting up a vicious cycle. Our work has further revealed a particularly important role for neutrophils and NETs in the thrombo-inflammatory disease manifestations that are relevant to not only APS and lupus but also other diseases, such as COVID-19 (19–21). We have also found that targeting NETosis through various mechanisms (neutrophil depletion, deoxyribonuclease, adenosine receptor agonists) mitigates thrombosis in APS models (18, 19, 22, 23). Safe agents that restrain NETosis in patients might therefore improve outcomes across various autoimmune and inflammatory diseases.

Natural herbs with antiinflammatory properties are potentially untapped resources in our search for agents that can combat pathogenic NETosis (24). We previously reported that a purified preparation of 6-gingerol, the most abundant bioactive phytochemical in ginger root, inhibited neutrophil phosphodiesterase (PDE) activity, boosting intracellular cyclic AMP (cAMP) levels and thereby counteracting neutrophil hyperactivity in mouse models of APS and lupus (22). In those experiments, purified 6-gingerol was delivered by intraperitoneal injection. To better understand the potential NET-inhibiting benefits of ginger in humans, studies using an orally administered ginger supplement are needed.

Here, we aimed to validate the effect of ginger on neutrophil activity, using an oral ginger extract and including healthy humans. Such a study can set the stage for the eventual clinical testing of ginger in patients with NET-driven autoimmune diseases, such as lupus, APS, rheumatoid arthritis, vasculitis, and even COVID-19.

## Results

### Ginger inhibits NETosis in response to various stimuli, including autoantibodies isolated from patients with lupus or APS.

**Figure 1:**
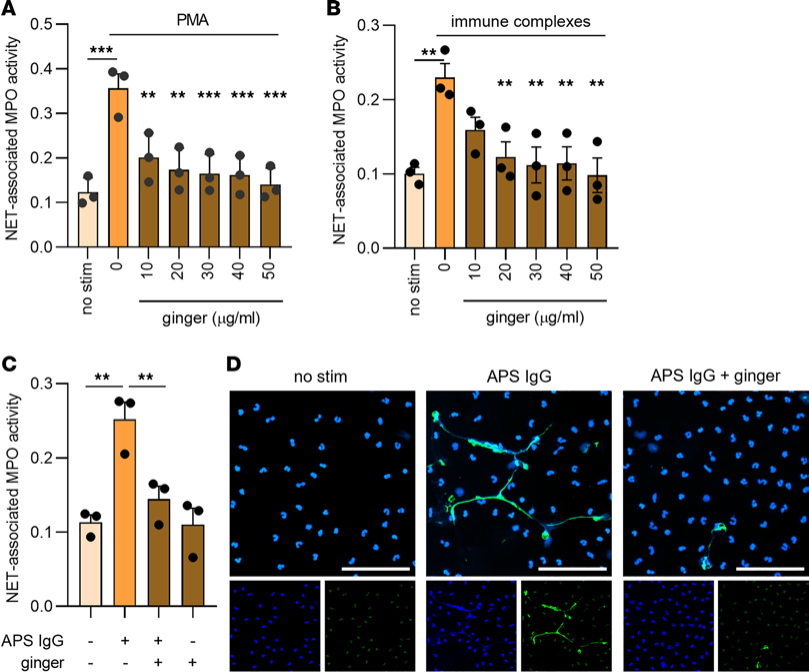
Ginger suppresses NETosis in response to various stimuli. Human neutrophils were isolated from healthy volunteers and then treated with various stimuli for 3 hours in the presence of different concentrations of ginger. NETosis was quantified by measuring the enzymatic activity of nuclease-liberated MPO. (**A** and **B**) Ginger extract dose response in the setting of PMA- and RNP/anti-RNP immune complex–mediated NETosis. (**C** and **D**) NETosis in response to APS IgG was quantified in the presence of 10 μg/mL ginger and assessed by either enzymatic activity of nuclease-liberated MPO (**C**) or immunofluorescence microscopy (**D**). Blue = DNA, green = extracellular neutrophil elastase, and scale bar = 100 μm. For panels **A**–**C**, mean and standard error of the mean (SEM) are presented for *n* = 3 independent experiments; **P < 0.01, and ***P < 0.001 by 1-way ANOVA corrected with Dunnett’s test.

We tested the ability of a whole-ginger extract, containing roughly 20% total gingerols (25), to inhibit the NETosis of neutrophils isolated from the blood of healthy human volunteers. NETosis was quantified, using an assay that we have described previously (19, 22, 23), via partial nuclease digestion (to release formed NETs into the neutrophil culture supernatant) followed by measuring the enzymatic activity of NET-associated myeloperoxidase (MPO). We found that ginger suppressed phorbol 12-myristate 13-acetate–induced (PMA-induced) NETosis in a concentration-dependent manner (Figure 1A). We then asked whether ginger would inhibit NETosis in response to autoimmune-relevant physiological stimuli. Indeed, ginger significantly attenuated both ribonucleoprotein (RNP)/anti-RNP immune complex– and APS IgG–mediated NETosis (Figure 1, B–D). Similar results were obtained when the whole-ginger extract was solubilized with phosphate-buffered saline (PBS, Figure 1), dimethylsulfoxide (DMSO), or polyethylene glycol (Supplemental Figure 1; supplemental material available online with this article; https://doi.org/10.1172/jci.insight.172011DS1).

### Ginger inhibits cAMP-specific PDE activity.

**Figure 2:**
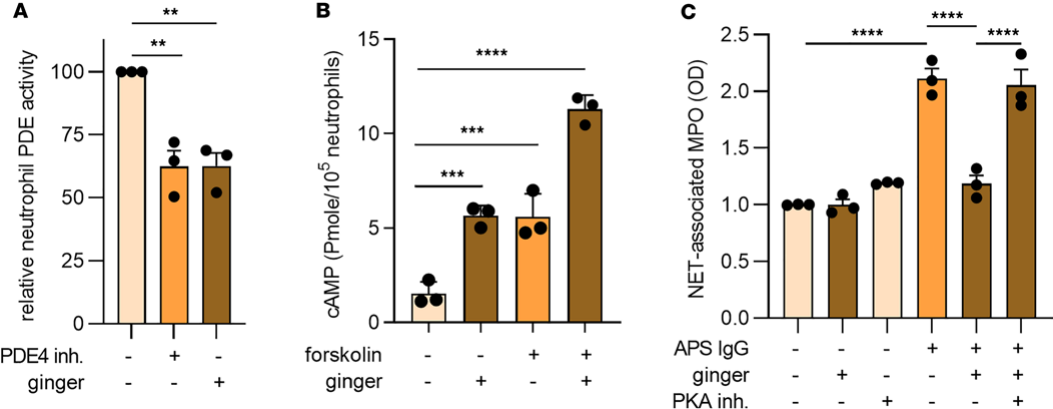
Ginger blocks PDE activity and increases cAMP levels. (**A** and **B**) Human neutrophils were treated with the solubilized ginger extract (10 μg/mL). Some samples were additionally treated with a synthetic PDE4 inhibitor (rolipram, 0.1 μM) or an adenylate cyclase activator (forskolin, 100 μM) as indicated. PDE activity (**A**) and cAMP levels (**B**) were then measured with kits as described in Methods. (**C**) Neutrophils were treated with APS IgG (10 μg/mL) in the presence or absence of a solubilized ginger extract (10 μg/mL) or a PKA inhibitor (KT 5720, 10 μM). NETosis was quantified by measuring the enzymatic activity of nuclease-liberated MPO. For all panels, mean and SEM are presented for *n* = 3–4 independent experiments; **P < 0.01, ***P < 0.001, and ****P < 0.0001 by 1-way ANOVA corrected with Dunnett’s test.

We previously found that 6-gingerol inhibits neutrophil PDE activity and thereby boosts neutrophil cAMP levels (22). Here, we verified the same is true in the setting of a solubilized whole-ginger extract. We found that the ginger extract reduced PDE activity by 40%, similar to the synthetic PDE4 inhibitor, rolipram (Figure 2A). In addition, ginger significantly increased intracellular cAMP concentrations when neutrophils were treated with either ginger alone or ginger in the context of the adenylate cyclase activator, forskolin (Figure 2B). Notably, the suppressive effects of ginger on NETosis could be mitigated by blocking protein kinase A (PKA) activity, a key downstream cAMP-dependent kinase (Figure 2C). Importantly, the PKA inhibitor did not have a direct effect on NETosis outside the context of ginger (Supplemental Figure 2). These data support and validate our previous findings that ginger attenuates NETosis in vitro through a mechanism that, at least partially, depends on inhibition of PDE activity, potentiation of cAMP levels, and resultant activation of PKA.

### Ginger attenuates APS IgG–mediated venous thrombosis in mice.

**Figure 3:**
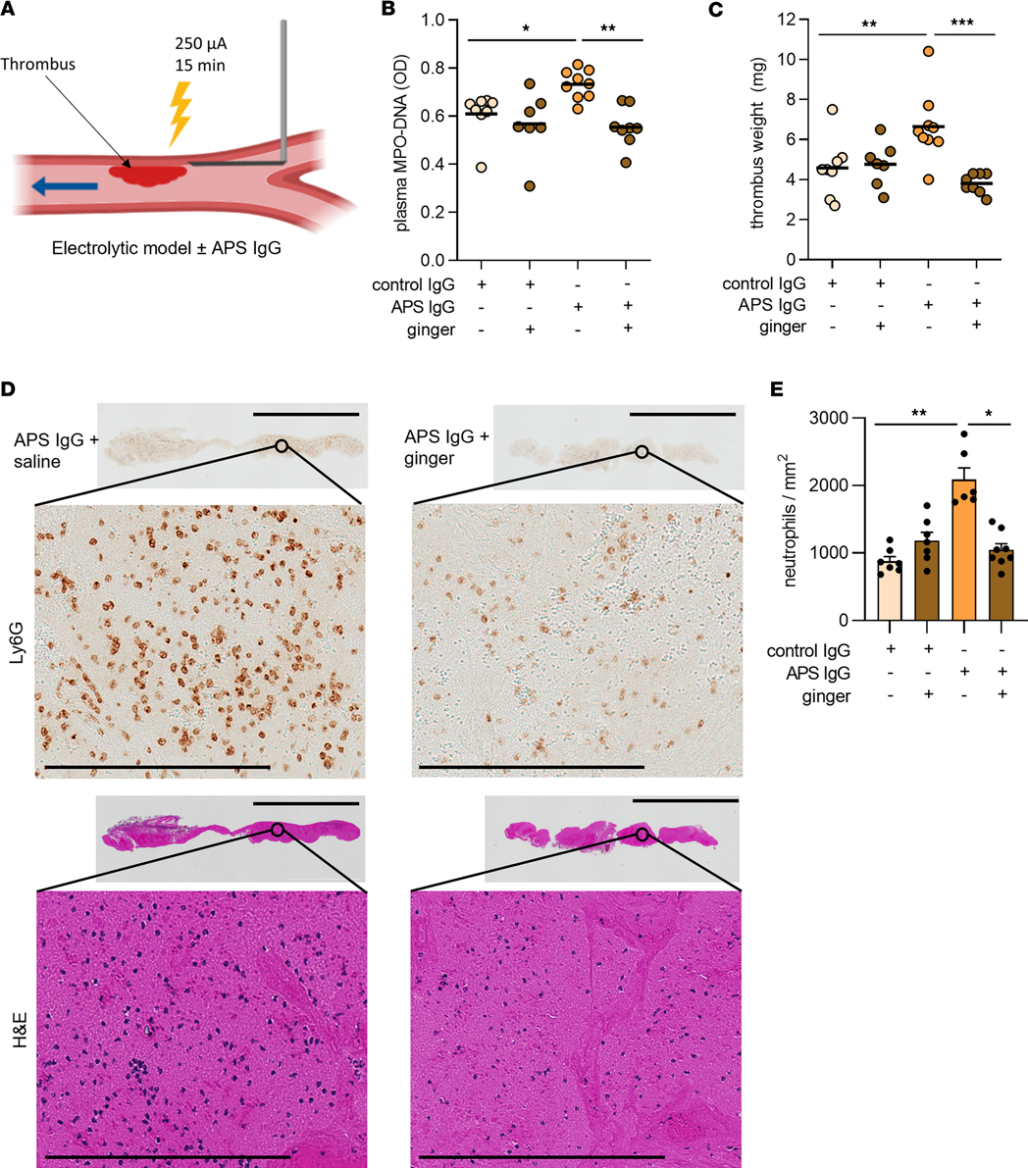
Ginger prevents APS IgG–mediated acceleration of venous thrombosis. (**A**) Schematic of the electrolytic model of venous thrombosis. Direct current results in the release of free radicals within the inferior vena cava, activating endothelial cells and initiating a thrombogenic environment in the setting of constant blood flow. (**B**) MPO-DNA complexes were measured in the serum of mice 24 hours after treatment with control IgG or APS IgG in the presence or absence of ginger (*n* = 7–9 mice/group). (**C**) Thrombus formation was assessed at 24 hours, and thrombus weight was measured (*n* = 7–9/group). (**D** and **E**) Thrombi (*n* = 6–8 mice/group) were sectioned and stained for the neutrophil marker Ly6G or with simple hematoxylin and eosin (H&E). Representative thrombus sections (scale bar, high power = 250 μm, low power = 3,000 μm) and neutrophils counts are presented. *P < 0.05, **P < 0.01, ***P < 0.001, and ****P < 0.0001 by 1-way ANOVA corrected with Dunnett’s test.

Since ginger suppressed APS IgG–triggered NETosis in vitro, we reasoned it might also reduce NETosis and downstream thrombosis in a mouse model of APS. We induced venous thrombosis using the electrolytic inferior vena cava model that we have described previously (Figure 3A) (19, 26). In this model, administration of APS IgG, as compared with control IgG, stimulates higher levels of circulating NETs and larger neutrophil-rich thrombi. As expected, administration of APS IgG increased serum NET levels (MPO-DNA complexes), which returned to baseline when mice received a ginger extract by daily oral gavage (Figure 3B). Furthermore, administering APS IgG, but not control IgG, increased thrombus weight, which again returned to control levels upon oral administration of ginger (Figure 3C). We sectioned 5 thrombi from each group and found that, as expected, neutrophils were the predominant nucleated cells within the thrombi of the APS mice. Neutrophil density was reduced in the thrombi of APS mice that also received ginger (Figure 3, D and E). In summary, these data demonstrate that ginger intake suppresses APS IgG–induced NETosis and venous thrombosis in vivo.

### Ginger attenuates lupus-relevant disease activity in mice.

**Figure 4:**
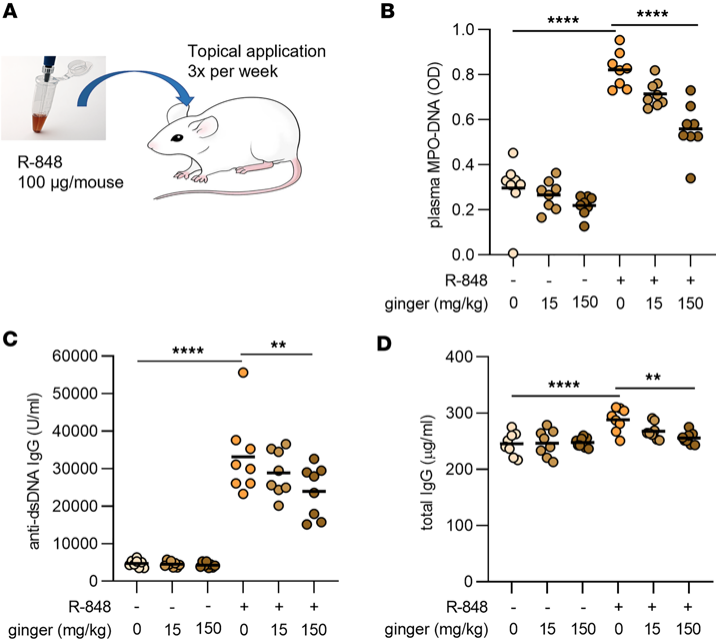
Ginger reduces NETosis and autoantibody formation in a lupus mouse model. (**A**) BALB/c mice were treated topically with a TLR7 agonist (R848) or vehicle (DMSO) for 6 weeks (3 times per week), and the ginger extract was incorporated into mouse chow throughout this period, targeting doses of approximately 15 and 150 mg/kg/d (*n* = 8 mice/group). (**B**) NET levels in serum were assessed by measuring MPO-DNA complexes. (**C** and **D**) Anti-dsDNA and total IgG levels in serum were assessed by ELISA. For all panels, the mean is presented as a horizontal line; **P < 0.01 and ****P < 0.0001 by 1-way ANOVA corrected with Dunnett’s test.

We next tested the efficacy of ginger consumption on disease activity in a lupus mouse model. In TLR7 agonist–treated (R848) mice (Figure 4A), oral intake of a ginger extract over 6 weeks resulted in a marked reduction in plasma NET levels, as indicated by decreased serum MPO-DNA complexes (Figure 4B). Lupus-relevant antibodies, including anti-dsDNA and total IgG, were also reduced (Figure 4, C and D). These data demonstrate that oral ginger intake reduces lupus-relevant NETosis and autoantibody formation in vivo.

### Administration of an oral ginger supplement to healthy volunteers boosts neutrophil cAMP and reduces NETosis.

**Figure 5:**
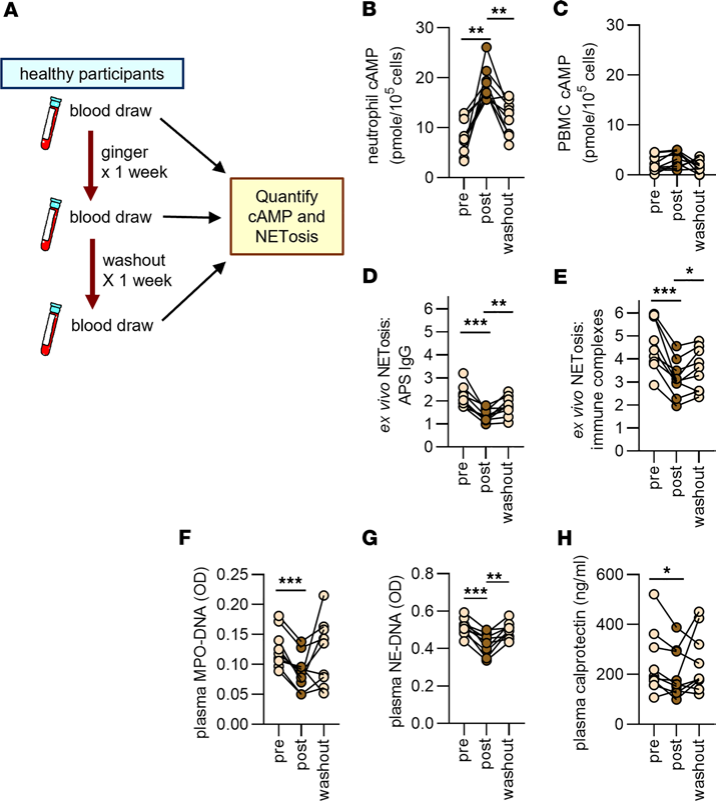
Administration of a ginger extract to healthy individuals boosts neutrophil cAMP and reduces ex vivo NETosis. (**A**) Nine participants who denied recent exposure to ginger supplements or ginger-containing foods consumed a 100 mg ginger extract once daily for 7 days. (**B** and **C**) Levels of cAMP were assessed in neutrophils and peripheral blood mononuclear cells (PBMCs). (**D** and **E**) Neutrophils were isolated from participants and stimulated with either APS IgG or RNP/anti-RNP immune complexes (both 10 μg/mL). NETosis was quantified by measuring extracellular DNA, and data are presented as fold-change in activation relative to unstimulated control neutrophils. (**F**–**H**) NET levels in plasma were assessed by measuring MPO-DNA complexes, neutrophil elastase–DNA (NE-DNA) complexes, or calprotectin. For all panels, *P < 0.05, **P < 0.01, ***P < 0.001 by 1-way ANOVA corrected with Dunnett’s test.

We designed a pilot clinical study to determine the extent to which the anti-neutrophil properties we observed in vitro and in mouse models might extend to humans (Figure 5A). Nine participants (University of Michigan) were recruited and consumed a ginger supplement (Pureveda Activ Digest, Aurea Biolabs) (27) at a dose of 100 mg (approximately 20 mg gingerols) once daily for 7 consecutive days. The average age of the participants was 27 years (range 18 to 38). Six were women and 3 were men. Six identified as White and 2 as Hispanic; race and ethnicity were not disclosed for the final participant. Blood was sampled (i) just before starting the ginger supplement, (ii) on day 7, and (iii) on day 14. Neutrophils, peripheral blood mononuclear cells (PBMCs), and plasma were isolated. Compared with pre-ginger samples, there was a significant increase in neutrophil cAMP after 7 days of ginger intake, which returned to near baseline 1 week after ginger consumption had ended (Figure 5B). Interestingly, this was a neutrophil-specific effect, with no significant change in cAMP found in PBMCs (Figure 5C). In parallel to neutrophil cAMP increases, there was a decrease in APS IgG– and RNP/anti-RNP immune complex–stimulated NETosis (Figure 5, D and E). Similarly, there was a reduction in plasma NET levels as measured by MPO-DNA complexes, neutrophil elastase–DNA complexes, or calprotectin (Figure 5, F–H). These data demonstrate that consumption of a ginger supplement by healthy individuals has the potential to alter neutrophil function in vivo, resulting in neutrophils less prone to NETosis.

### Replication of the neutrophil-specific effects of oral ginger in healthy volunteers.

**Figure 6:**
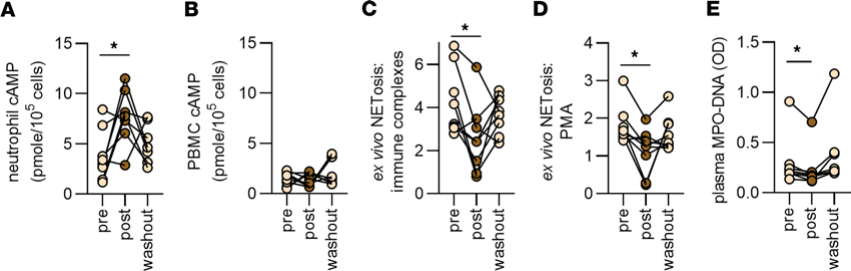
Administration of a ginger extract to healthy individuals at a second site boosts neutrophil cAMP and reduces ex vivo NETosis. Eight participants who denied recent exposure to ginger supplements or ginger-containing foods consumed a 100 mg ginger extract once daily for 7 days. (**A** and **B**) Levels of cAMP were assessed in neutrophils and PBMCs. (**C** and **D**) Neutrophils were isolated from participants and stimulated with either RNP/anti-RNP immune complexes (10 μg/mL) or PMA (100 nM). NETosis was quantified by measuring extracellular DNA, and data are presented as fold activation relative to unstimulated control neutrophils. (**E**) NET levels in plasma were assessed by measuring MPO-DNA complexes. For all panels, *P < 0.05 by 1-way ANOVA corrected with Dunnett’s test.

To validate our findings in healthy volunteers, we replicated the same pilot study design described above at a second site (University of Colorado) in 8 additional participants. The average age of the participants was 31 years (range 24 to 51). Four were women and 4 were men; 7 identified as White and 1 as Asian. After 7 days of ginger intake, similar to our findings in the first cohort, we found a neutrophil-specific increase in cAMP that was not found in PBMCs (Figure 6, A and B). Parallel to neutrophil cAMP increases, there was a decrease in NETosis upon stimulation with either RNP/anti-RNP immune complexes or PMA (Figure 6, C and D). There was also a reduction in plasma NET levels as measured by MPO-DNA complexes (Figure 6E).

## Discussion

We found that oral administration of ginger modulates neutrophils and NETosis in autoimmune mouse models and healthy humans. In mice, intake of a ginger extract (either by oral gavage or mixed with chow) reduced NETosis in models of APS and lupus. This was accompanied by a decrease in disease-relevant phenotypes, including thrombosis in the setting of APS and autoantibody formation in the setting of lupus. In 2 separate cohorts of healthy humans, daily consumption of a ginger supplement resulted in a neutrophil-specific increase in cAMP with no impact on PBMC cAMP. Parallel to the increase in neutrophil cAMP, there was a decrease in both stimulated NETosis and circulating NET levels following consumption of ginger.

The mechanistic details reported here are in line with our previous work that relied on a purified preparation of 6-gingerol (22). Here, we found that the solubilized ginger extract antagonized neutrophil PDE activity. The result was increased neutrophil intracellular cAMP levels, which associated with blunted NETosis by human neutrophils in vitro. Such data extend recent studies that have reported a role for ginger extracts, and specifically 6-gingerol, as inhibitors of cAMP-specific PDE activity (28, 29). Importantly, the suppressive effects of ginger on NETosis could be mitigated by blocking PKA activity, a key downstream cAMP-dependent kinase. The fact that increasing neutrophil cAMP and activating PKA would be beneficial for disease activity in mice aligns well with our previous work demonstrating the potential therapeutic target of this pathway in APS and lupus models with synthetic PDE4 inhibitors (22). Indeed, PDE inhibitors are attractive therapeutic targets for chronic inflammatory diseases (30, 31). Specifically, there has been targeted drug development against PDE4, the predominant isotype expressed by leukocytes (32, 33). For example, apremilast is a selective PDE4 inhibitor currently used in the clinic in the context of psoriasis, psoriatic arthritis, and Behçet’s disease (34). Roflumilast is also another PDE4 inhibitor approved for the treatment of inflammatory airway diseases such as asthma and chronic obstructive pulmonary disease, while crisaborole is approved for atopic dermatitis (35).

Through the pilot clinical trial, we found that the properties of the ginger extract that were observed in vitro and in mice are likely to extend to humans. Furthermore, the rigor of these findings was increased by replication of the results at a second site. There are myriad ginger extracts/supplements available to consumers. Here, we performed preliminary studies in mice and humans using a commercially available whole-ginger extract (Pureveda Activ Digest) provided to us for research purposes by Aurea Biolabs. This ginger supplement is manufactured to include relatively high concentrations of gingerols (approximately 20%) (25) but with a recommended daily dose of gingerols (20–40 mg/d) that aligns well with other available ginger supplements on the market. Some examples include Pure Encapsulations Ginger Extract (25–100 mg/d), Doctor’s Best High Potency Ginger Root Extract (25 mg/d), and Swanson Ginger Root Extract (40 mg/d). The extent to which these different preparations may differ in their bioavailability does, however, require further study.

Acknowledging these data in healthy individuals, the impact of ginger supplementation on the neutrophils of patients with inflammatory diseases such as APS and lupus has not yet been tested. Further, the extent to which this modulation of neutrophils could have an impact on the ability of neutrophils to respond to infection is a topic that should receive attention in future studies. Finally, although some human trials suggest improved arthritis symptoms and reduced thrombotic risk with ginger supplementation, mechanistic studies investigating NETosis have never been performed to date. Future clinical studies are needed to investigate this potential adjuvant therapeutic intervention that targets a pathogenic mechanism (NETosis) shared by various autoimmune diseases.

## Methods

### Ginger extract.

A ginger extract (Ginactiv) was provided to us for research purposes by Aurea Biolabs (Kerala, India) in powder form for the in vitro and mouse studies and in typical capsule/supplement form for the human studies (27). For in vitro experiments, the powdered ginger extract was solubilized in PBS, and any remaining insoluble material was filtered out. For the APS thrombosis model, the ginger extract was solubilized in normal saline and administered to mice by oral gavage. For the lupus model, the ginger extract was incorporated into mouse chow by Teklad Laboratory Animal Diets and provided to mice ad libitum. For the clinical trial, a commercially available ginger supplement containing Ginactiv, branded as Pureveda Activ Digest 100 mg, was provided by Aurea Biolabs.

### Purification of IgG.

IgG was purified from healthy volunteers or patients with APS or lupus with the Protein G Agarose Kit (Pierce) following the manufacturer’s instructions, as previously described (19). Briefly, serum or plasma was diluted in IgG binding buffer and passed through a Protein G Agarose column. IgG was then eluted with 0.1 M glycine and neutralized with 1 M Tris. This was followed by overnight dialysis against PBS at 4°C. IgG purity was verified with Coomassie staining, and concentrations were determined by BCA protein assay (Pierce) according to the manufacturer’s instructions. All IgG samples were determined to be free of detectable endotoxin by the Pierce LAL Chromogenic Endotoxin Quantitation Kit according to the manufacturer’s instructions.

### Human neutrophil purification and NETosis assays.

Blood from healthy volunteers was collected into heparin tubes by standard phlebotomy techniques. The anticoagulated blood was then fractionated by density-gradient centrifugation using Ficoll-Paque Plus (GE Healthcare, now Cytiva). Neutrophils were further purified by dextran sedimentation of the red blood cell layer before lysing residual red blood cells with 0.2% sodium chloride. Neutrophil preparations were at least 95% pure, as verified by flow cytometry and nuclear morphology. To assess NETosis, neutrophils were resuspended in RPMI medium (Gibco) supplemented with 0.5% bovine serum albumin (BSA, MilliporeSigma) and 0.5% fetal bovine serum (Gibco), which had been heat-inactivated at 56°C. Neutrophils (1 × 10^5^/well) were cultured in 96-well plates at 37°C with 100 nM PMA (MilliporeSigma), 10 μg/mL RNP/anti-RNP immune complexes (36), or 10 μg/mL APS IgG. RNP/anti-RNP immune complexes were formed by mixing IgG purified from 3 individuals with lupus and anti-RNP positivity with SmRNP (Arotec) (36). APS IgG was pooled from 3 patients with triple-positive APS including high-titer anti–beta-2 glycoprotein I antibodies. In some cases, cultures were also supplemented with different concentrations of a solubilized ginger supplement (Aurea Biolabs, as described above) or 10 μM PKA inhibitor, KT 5720 (Tocris). After 3 hours in culture, NET-associated MPO activity was measured as follows. First, the culture medium was discarded (to remove any soluble MPO) and replaced with 100 μL of RPMI supplemented with 10 U/mL Micrococcal nuclease (Thermo Fisher Scientific). After 10 minutes at 37°C, digestion of NETs was stopped with 10 mM EDTA. Next, supernatants were transferred to a V-shaped, 96-well plate and centrifuged at 350*g* for 5 minutes at room temperature to remove debris. Supernatants were then transferred into a new plate. An equal volume of 3,3′,5,5′-tetramethylbenzidine (3,3′,5,5′-TMB) substrate (1 mg/mL, Thermo Fisher Scientific) was added to each well to measure MPO activity. After 10 minutes of incubation in the dark, the reaction was stopped by adding 50 μL of 1 mM sulfuric acid. Absorbance was measured at 450 nm using a BioTek Cytation 5 Cell Imaging Multi-Mode Reader. In some cases, NETosis was assessed by a SYTOX Green–based assay (Thermo Fisher Scientific), which detects extracellular DNA. Neutrophils were cultured as above in 96-well black microplates (with clear, flat bottoms) at 37°C. After 3 hours, SYTOX Green was added to a final concentration of 0.2 μM and incubated for an additional 10 minutes. Fluorescence was quantified at excitation and emission wavelengths of 485 nm and 520 nm, respectively, using a Cytation 5 Cell Imaging Multi-Mode Reader (BioTek) with the following settings: Light Source: Xenon Flash; Lamp Energy: High; Extended Dynamic Range Read Speed: Normal; Delay: 10 ms; Measurements/Data Point: 10; Read Height: 7 mm.

### Immunofluorescence microscopy.

For immunofluorescence microscopy, 1.5 × 10^5^ neutrophils were seeded onto coverslips coated with 0.001% poly-l-lysine (MilliporeSigma) and fixed with 4% paraformaldehyde for 15 minutes at room temperature. Blocking was with 1% BSA overnight at 4°C. The primary antibody was against neutrophil elastase (Abcam catalog ab21595, diluted 1:100), and the FITC-conjugated secondary antibody was from Southern Biotech (catalog 4052-02, diluted 1:250). DNA was stained with Hoechst 33342 (Invitrogen). Images were collected with a Cytation 5 Cell Imaging Multi-Mode Reader.

### Measurement of PDE activity.

Human neutrophils (1 × 10^7^) were washed twice with cold PBS and pelleted by centrifugation at 2,500*g* for 5 minutes at room temperature. The cell pellet was resuspended in 100 μL of RIPA buffer (MilliporeSigma, R0278) supplemented with protease inhibitors (Roche Diagnostics, 35440400) for 15 minutes on ice. The mixture was centrifuged at 14,000*g* for 5 minutes at room temperature to clear cell debris. The activity of PDE was measured using the Bridge-It cAMP-PDE assay kit (Mediomics, catalog PD-1016). The supernatant was mixed with the reaction mixture according to the manufacturer’s instructions in the presence or absence of 10 μg/mL of ginger extract or 0.1 μM PDE4 inhibitor rolipram and allowed to proceed for 1 hour at 37°C. The reaction was stopped, and the assay solution was added. After 30 minutes at 37°C, fluorescence was measured with a Synergy HT Multi-Mode Microplate Reader (BioTek) at excitation 480 nm and emission 520 nm. Relative activity was calculated and normalized to mean control values.

### Measurement of intracellular cAMP.

cAMP levels in human neutrophils were measured using the Bridge-It cAMP Designer fluorescence assay kit (Mediomics, catalog 122934). Briefly, neutrophils (1 × 10^5^) were washed twice with PBS and resuspended in 100 μL Krebs-Ringer bicarbonate buffer (without IBMX). Neutrophils were incubated at room temperature for 30 minutes in the presence or absence of 10 μg/mL ginger extract. Neutrophils were then stimulated with 100 μM forskolin for 10 minutes. Samples were centrifuged at 12,000*g* for 2 minutes at room temperature, and supernatants were discarded. The cAMP designer assay solution was then added to the cell pellet and carefully transferred to a 96-well, black-side, clear-bottom plate. The plate was incubated at room temperature for 30 minutes before measuring fluorescence with a Synergy HT Multi-Mode Microplate Reader at excitation 480 nm with bandpass 20 and emission 540 nm with bandpass 40.

### Animal housing and treatments.

Mice were purchased from The Jackson Laboratory, housed in a specific pathogen–free barrier facility, and fed standard chow. Male C57BL/6 mice (10–13 weeks) were used for venous thrombosis experiments, and the ginger extract (Aurea Biolabs) was administered by daily oral gavage at a dose 150 mg/kg daily for 7 days prior to surgery. Female BALB/c mice (9 weeks old) were used for the model of lupus induced by R848 with the ginger extract incorporated into mouse chow by Teklad Laboratory Animal Diets. The ginger extract was premixed with a portion of the 8604 diet and then added to the remainder of the base diet in the mixer. Water was added as a pelleting aid and mixed for approximately 10 minutes prior to being pressed into pellets. Pellets were dried at 50°C for 8 hours to remove the added water and then cooled, dried, and packaged. Since each mouse weighed approximately 25 grams and likely took in approximately 4 grams of food per day, the intention was for mice to receive either 15 mg/kg/d or 150 mg/kg/d of ginger. Given the faster metabolic rate of mice compared with humans, allometric scaling suggests the 15 mg/kg/d dose in mice would be roughly equivalent to a dose of 1.2 mg/kg/d in humans, while the 150 mg/kg/d dose would be roughly equivalent to 12 mg/kg/d (37).

### In vivo venous thrombosis.

To model venous thrombosis, we employed an electrolytic inferior vena cava (IVC) model as previously described (19, 26). Briefly, after exposure of the IVC, any lateral branches were ligated using a 7-0 Prolene suture (back branches remained patent). Next, the exposed end of a 30-gauge, silver-coated copper wire (KY-30-1-GRN, Electrospec) was placed inside a 25-gauge needle and inserted into the IVC, where it was positioned against the anterior wall (anode). Another wire was implanted subcutaneously, completing the circuit (cathode). A constant current of 250 μA was applied for 15 minutes. The current was supplied by a voltage-to-current converter as described previously (26). After the removal of the needle, the abdomen was closed. Before recovery from anesthesia, mice received a single intravenous injection of either control or APS IgG (500 μg) retro-orbitally. Blood was collected via cardiac puncture and the mice were humanely euthanized 24 hours later. Thrombi were carefully excised from the IVC and weighed before fixing in neutral buffered formalin.

### Quantification of MPO-DNA complexes.

MPO-DNA complexes were quantified similarly to what has been previously described (38). This protocol used several reagents from the Cell Death Detection ELISA kit (Roche). First, a high-binding EIA/RIA 96-well plate (Costar) was coated overnight at 4°C with anti-human MPO antibody (Bio-Rad 0400-0002), diluted to a concentration of 0.5 μg/mL in coating buffer (Cell Death kit). Next, the plate was washed 3 times with wash buffer (0.05% Tween 20 in PBS) and then blocked with 1% BSA in PBS for 1 hour at room temperature. Next, the plate was washed 3 times before incubating for 1 hour at room temperature with 1:500 mouse serum in the blocking buffer. Next, the plate was washed 5 times and then incubated for 1 hour at room temperature with 1× anti-DNA antibody (HRP conjugated; Cell Death kit) diluted 1:100 in blocking buffer. After 5 more washes, the plate was developed with 3,3′,5,5′-TMB substrate followed by a 2N sulfuric acid stop solution. Absorbance was measured at a wavelength of 450 nm with a Synergy HT Multi-Mode Microplate Reader (BioTek). Data were normalized to an in vitro–prepared NET standard and included on every plate.

### Thrombus sectioning and immunohistochemistry.

Isolated thrombi were fixed in formalin, embedded in paraffin, and sectioned according to standard protocols. For all thrombi with adequate tissue remaining after processing, immunohistochemical staining was performed in the University of Michigan Rogel Cancer Center Histology Core on a Dako Autostainer Link 48 (Agilent). Heat-induced epitope retrieval of deparaffinized sections was with EnVision FLEX Target Retrieval Solution, Low pH (Dako Omnis, Agilent), according to the manufacturer’s instructions. After blocking with peroxidase blocker for 5 minutes, the primary antibody was Rat Anti-Mouse Ly6G (BD Biosciences, 551459) at 1:500 for 30 minutes at room temperature. This was followed by the ImmPRESS HRP Goat Anti-Rat IgG Polymer Detection Kit (Vector Laboratories) with diaminobenzidine as the chromogen. Images of thrombi were captured with a Cytation 5 Cell Imaging Multi-Mode Reader. ImageJ (NIH) was used to quantify neutrophil density by counting the number of Ly6G-positive cells in a representative area of each thrombus section.

### R848 treatment.

Female BALB/c mice (9 weeks) were treated with the TLR7 agonist resiquimod (R848, Enzo Life Science) as previously described (39) with slight modifications. The epicutaneous application was to the ear 3 times per week with 100 μg R848 dissolved in 8 μL DMSO. Serum and tissues were collected after 6 weeks of treatment.

### Quantification of anti-dsDNA and total IgG.

Kits for mouse anti-dsDNA (catalog 5120) and mouse total IgG (catalog 6320) were purchased from Alpha Diagnostic International and performed according to the manufacturer’s instructions.

### Human research.

To participate in this project, participants had to be healthy (not meeting exclusion criteria) and 18 years of age or older. Exclusionary criteria were the following: active pregnancy or breastfeeding; recent pregnancy (within the last 6 months); experiencing active infection; history of hemoglobin < 12 g/dL within the last 6 months; current diagnosis of diabetes, cardiovascular disease, autoimmune disease, or active cancer; taking a regular chronic medication (other than for mental health or contraception); having a BMI greater than 30; current use of ginger or turmeric (either dietary or as a supplement); and ginger allergy. Individuals of childbearing potential were required to use an effective form of contraception or have a negative pregnancy test at screening.

At the University of Michigan (IRB approval HUM00209123), 9 healthy participants consumed a ginger supplement (Pureveda Activ Digest, Aurea Biolabs) 100 mg once daily. Supplement administration was for 7 (±2) days, and participants were followed for 7 (±2) additional days after their last dose of treatment. The total duration of the study was 14 (±4) days. Blood was sampled just prior to starting the supplement and then again on day 7 and day 14. At all time points, neutrophils were isolated to determine neutrophil cAMP content and ex vivo NETosis in response to either APS IgG or RNP/anti-RNP immune complexes. Plasma samples were used to test for NET markers. This pilot clinical trial was replicated at the University of Colorado (IRB approval 22-0230) in 8 healthy participants meeting the same inclusion and exclusion criteria and following the same administration and blood sampling strategy.

### Quantification of neutrophil elastase–DNA complexes.

This protocol used several reagents from the Cell Death Detection ELISA kit (Roche). First, a high-binding EIA/RIA 96-well plate (Costar) was coated overnight at 4°C with anti-human neutrophil elastase antibody (MilliporeSigma, 481001), diluted to a concentration of 5 μg/mL in coating buffer (Cell Death kit). Next, the plate was washed 3 times with wash buffer (0.05% Tween 20 in PBS) and then blocked with 1% BSA in PBS for 1 hour at room temperature. Next, the plate was washed 3 times before incubating overnight at 4°C with 10% human plasma in the blocking buffer. Next, the plate was washed 3 times and then incubated for 1 hour at room temperature with 1× anti-DNA antibody (HRP conjugated; Cell Death kit) diluted 1:100 in blocking buffer. After 5 more washes, the plate was developed with 3,3′,5,5′-TMB substrate followed by a 2N sulfuric acid stop solution. Absorbance was measured at a wavelength of 450 nm with a Synergy HT Multi-Mode Microplate Reader. Data were normalized to an in vitro–prepared NET standard and included on every plate.

### Quantification of S100A8/A9 (calprotectin).

Calprotectin was measured with the Human S100A8/S100A9 Heterodimer DuoSet ELISA (DY8226-05, R&D Systems) according to the manufacturer’s instructions.

### Statistics.

Groups were compared by 1-way ANOVA corrected for multiple comparisons. *P* values less than 0.05 were considered statistically significant.

### Study approval.

All studies in animals and humans were reviewed and approved by an appropriate review board. Specifically, human studies were approved by the University of Michigan IRB (Ann Arbor, Michigan, USA) and the University of Colorado IRB (Aurora, Colorado, USA). Animal studies were approved by the University of Michigan IACUC (Ann Arbor, Michigan, USA).

### Data availability.

All raw data are provided in the accompanying Supporting Data Values file.

## Author contributions

RAA, VCM, MZ, and CER conducted experiments and analyzed data. CS, CKH, and KAS obtained regulatory approvals and recruited healthy volunteers. RAA, VCM, JSK, and MKD designed the study and analyzed the data. All authors contributed to drafting the manuscript and approved the final submitted version.

## Supplementary Material

Supplemental data

Supporting data values

## Version 1 09/22/2023

Electronic publication
