# Supplementary material for: Ginger intake suppresses neutrophil extracellular trap formation in autoimmune mice and healthy humans
Source: JCI Insight. 2023 Sep 22;8(18):e172011. doi: 10.1172/jci.insight.172011 (PMC10561719; doi:10.1172/jci.insight.172011)
Supplement: Supplemental data [file jciinsight-8-172011-s073.pdf]

SUPPLEMENATRY INFORMATION

Ginger intake suppresses neutrophil extracellular trap formation in autoimmune mice and healthy humans

Ali *et al.*

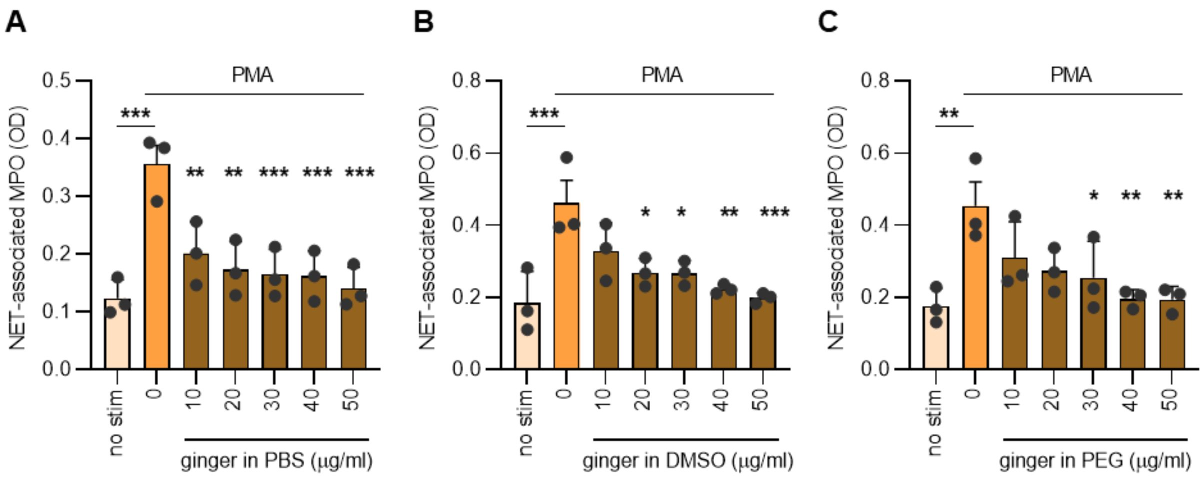

Supplementary Figure 1: Similar effects on NETosis upon solubilization of a ginger extract with different solvents. A-C, Human neutrophils were isolated from healthy volunteers and then treated with phorbol 12-myristate 13-acetate (PMA) for 3 hours in the presence of different concentrations of a ginger extract solubilized with PBS (A), dimethylsulfoxide (DMSO, B) or polyethylene glycol (PEG, C). NETosis was quantified by measuring the enzymatic activity of nuclease-liberated myeloperoxidase (MPO). For all panels, mean and standard error of the mean (SEM) are presented for n=3 independent experiments; *$p < 0.05$, **$p < 0.01$, and ***$p < 0.001$ as compared with the PMA alone group by one-way ANOVA corrected with Dunnett's test.

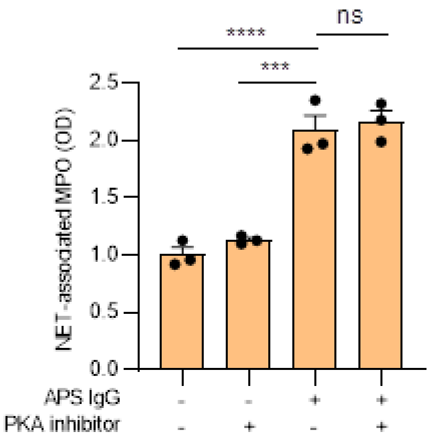

Supplementary Figure 2: PKA inhibition does not have a direct effect on NETosis. Neutrophils were treated with APS IgG (10 µg/ml) in the presence or absence of a PKA inhibitor (KT 5720, 10 µM). NETosis was quantified by measuring the enzymatic activity of nuclease-liberated myeloperoxidase (MPO). Mean and standard error of the mean (SEM) are presented for n=3 independent experiments; ***$p < 0.001$, and ****$p < 0.0001$ by one-way ANOVA corrected with Dunnett's test; ns=not significant.
